# Metabolomics analysis of baicalin on ovalbumin-sensitized allergic rhinitis rats

**DOI:** 10.1098/rsos.181081

**Published:** 2019-02-20

**Authors:** Saizhen Chen, Guirong Chen, Sheng Shu, Yubin Xu, Xiande Ma

**Affiliations:** 1 Taizhou Central Hospital (Taizhou University Hospital), Taizhou 318000, People's Republic of China; 2 Liaoning University of Traditional Chinese Medicine, Shenyang 110847, People's Republic of China

**Keywords:** metabolomics, baicalin, allergic rhinitis

## Abstract

Allergic rhinitis (AR) is a global health problem that appears in all age groups and affects approximately 15–30% of people. Baicalin has been used for the treatment of various allergic diseases, including AR. However, the metabolic mechanisms of AR and baicalin against AR have not been systematically studied. Here, ovalbumin-sensitized AR rats were used as a model, and animal behaviour, histological analysis, enzyme-linked immunosorbent assay (ELISA) and metabolomics were used to elucidate the mechanism of baicalin for AR. The results indicated that baicalin has a protective effect on AR rats by inhibiting the release of immunoglobulin E (IgE), histamine, interleukin-1 beta (IL-1β), interleukin-4 (IL-4), interleukin-6 (IL-6) and tumour necrosis factor alpha (TNF-α). In addition, ovalbumin-induced AR included modulation of arachidonic acid, leukotriene A4 (LTA4), leukotriene B4 (LTB4), α-ketoglutaric acid, phosphatidylcholine PC (20 : 4/0 : 0), PC (16 : 0/0 : 0), citric acid, fumarate, malate, 3-methylhistidine, histamine and other amino acids that are involved in arachidonic acid, histidine metabolism, the TCA cycle and amino acid metabolism. Thus, AR could be alleviated or reversed by baicalin.

## 1. Introduction

Allergic rhinitis (AR) is a type of chronic inflammation in the respiratory tract [1]. It is a very common disease of nasal mucosa, triggered by the interaction of specific immunoglobulin E (IgE) and ubiquitous environmental proteins in sensitized patients [2]. The main symptoms of AR are sneezing, rhinorrhea, itching and nasal congestion; these symptoms are usually accompanied by other related symptoms such as nasal mucosa swelling [3,4]. Currently, AR appears in all age groups, affects a large part of the global population and is a major public health burden [5]. Epidemiologic data have shown that AR affects nearly 15–30% of people [6,7].

Current studies have predominantly concentrated on cytokines, chemokines such as interleukin-1 beta (IL-1β), tumour necrosis factor alpha (TNF-α) and interleukin-4 (IL-4), which are involved in the pathogenesis and process of allergic reactions during AR. However, the effective metabolites related to AR remain unclear [8,9]. It is imperative to explore potential metabolites to systematically describe the mechanism of AR.

Recently, the use of natural products for treating AR has been increasing worldwide; many prior trials have evaluated the effectiveness of herbal medicines for AR [10]. Baicalin, a flavonoid compound isolated from *Scutellaria baicalensis* Georgi, has been shown to have multiple pharmacological activities, including anti-allergic, anti-inflammatory and antioxidant effects [2]. Based on its anti-allergic and anti-inflammatory activity, baicalin is widely used to treat various allergic and inflammatory diseases, including asthma, atopic dermatitis and AR [11]. However, there is little information about the metabolic mechanism of baicalin on ovalbumin (OVA)-induced AR in Sprague-Dawley (SD) rats.

In this study, an untargeted metabolomics technique was used to evaluate the metabolic mechanism of AR SD rats, and the systematic mechanism of baicalin against OVA-induced AR was explored. This study might provide new insights to understand AR as well as the potential application of baicalin as a drug for AR treatment.

## 2. Material and methods

### 2.1. Reagents

Baicalin was purchased from the National Institute for the Control of Food and Drug (Beijing, China). HPLC-grade methanol, acetonitrile and formic acid were purchased from Merck (Germany). The enzyme-linked immunosorbent assay (ELISA) kits for IgE, histamine, IL-4, IL-1β, interleukin-6 (IL-6) and TNF-α were purchased from Uscn Life Science Inc. (Wuhan, China). OVA, leucine-enkephalin and all the authentic standards were purchased from Sigma-Aldrich (St. Louis, MO, USA).

### 2.2. Animal experiments and sample collection

Male SD rats (200 ± 20 g) were obtained from Liaoning Changsheng Biotechnology Co. Ltd. (Liaoning, China). The animals were maintained under specific pathogen free (SPF) laboratory conditions and received human care according to the guidelines of the Local Institutes of Health guide for the care and use of laboratory animals.

All SD rats were randomly divided into three groups, with 10 rats in each group: a control group, model group and baicalin group. First, the model group and baicalin group were sensitized by OVA using a standard protocol as described in a previous study [12]. Briefly, the rats were sensitized by OVA via injection on days 1, 5 and 10; from days 15 to 21, the sensitized rats were intranasally challenged by daily droppings with OVA. After the AR models were successfully established, the rats in the baicalin group were orally administered baicalin for 10 days from days 22 to 31 at a dose of 200 mg kg^−1^, as previously reported [2]. The rats in the model group received saline on the same days. The control group rats received saline alone on the same schedule.

The numbers of sneezes and nose scratching were counted for 15 min after 2 h of the last administration. Then, after isoflurane anaesthesia, the rats were sacrificed by drawing all the blood from the abdominal aorta to obtain blood samples, and the nasal mucosa was separated and fixed in 4% paraformaldehyde at room temperature. The blood samples were centrifuged at 3000*g* for 10 min at 4°C to obtain serum, which was then stored at −80°C until analysis.

### 2.3. Histological analysis

The nasal tissues were dehydrated, embedded in paraffin, sectioned at 4 µm thickness, stained with haematoxylin and eosin and then analysed with light microscopy.

### 2.4. Measurement of IgE, histamine, IL-1β, IL-4, IL-6 and TNF-α levels in the serum

IgE, histamine, IL-1β, IL-4, IL-6 and TNF-α levels in the serum were determined using ELISA kits according to the manufacturer's instructions.

### 2.5. Metabolomics

#### 2.5.1. Sample preparation and UPLC/Q-TOF–MS/MS analysis

The method was performed as previously described with minor modifications [13]. Serum (200 µl) was added to 600 µl acetonitrile, vortex-mixed for 30 s and centrifuged at 12 000*g* for 5 min to precipitate the proteins. Next, 600 µl of supernatant was collected and dried with vacuum drying and centrifuged at room temperature. Then, the dried residue was reconstituted with 100 µl of an acetonitrile-water solution (1 : 1, v/v). After centrifugation at 12 000*g* for 5 min, an aliquot of 3 µl was injected for UPLC/Q-TOF–MS/MS analysis.

A Waters Acquity UPLC system was used to perform the analysis. The chromatographic column was an HSS T3 column (2.1 × 100 mm, 1.7 µm, Waters, USA), and the column temperature was maintained at 40°C. The mobile phase was methanol (A) and 0. 1% formic acid (B), and the gradient elution conditions are described as follows: 0 → 7 min: 0% → 60%A; 7 → 10 min: 60% → 100%A; 10 → 14 min: 100%A; 14 → 16 min: 100% → 0%A; Curve: 6.

Mass spectrometry was performed on a Waters Xevo G2 Q-TOF quadrupole accelerated time of flight mass spectrometer in positive and negative ion mode with an electrospray ionization (ESI) source. The desolvation gas was 800 l h^−1^, the desolvation temperature was 400°C, the cone gas was 50 l h^−1^ and the temperature of the ion source was 110°C. The capillary voltage, cone voltage, scanning time and collision energy were set as 3.0 kV, 45 V, 1 s and 6 eV, respectively. The spectra were collected every 0.2 s alternately with the interval at 0.02 s, and the mass range was from 50 to 1200 Da. The LockSpray^TM^ was set to *m/z* 556.2771([M + H]) and 554.2615[M − H] for leucine-enkephalin.

#### 2.5.2. Data processing

The data processing was performed as previously described. Briefly, the raw MS spectra were analysed using MarkerLynx v. 4.1 (Waters Corp., UK). Then, the processed data list was exported and processed by orthogonal partial least-squares discriminant analysis (OPLS-DA) using the software package Simca v. 14.1 (Umetrics, Sweden).

### 2.6. Statistical analysis

All values were expressed as the mean ± s.d. The significance of differences between the means of control, model and baicalin groups was compared through one-way ANOVA using the Statistical Package for Social Sciences program (SPSS 20.0, USA). The significance threshold was set at *p* < 0.05 for this test.

## 3. Results and discussion

### 3.1. Animal behaviour study

**Figure 1.**
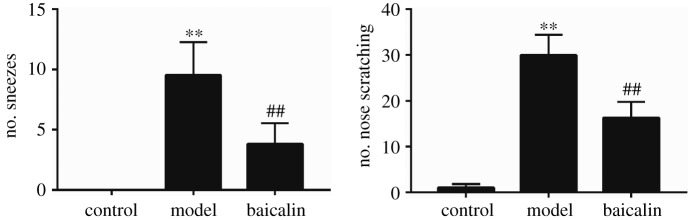
The nasal sneezes and scratching numbers of rats in each group. Compared to control group, *p < 0.05, **p < 0.01; Compared to model group, #*p* < 0.05, ##*p* < 0.01.

The nasal symptoms of rats were recorded for 15 min. The numbers of nasal sneezes and scratching significantly increased in the model group and significantly decreased in the baicalin group. The scores for the groups are shown in figure 1. The results indicated that the OVA-sensitized rat model was successfully established, and the nasal symptoms could be inhibited by baicalin at a dose of 200 mg kg^−1^.

### 3.2. Histological analysis

**Figure 2.**
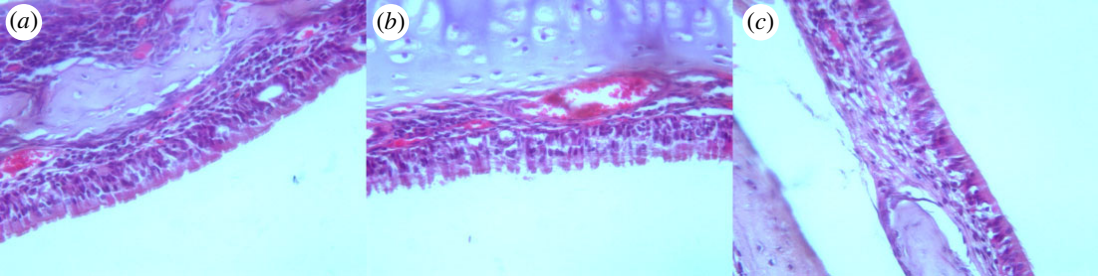
The pathological changes of nasal mucosa of rats in each group by histological analysis. (*a*) Control group; (*b*) model group; (*c*) baicalin group.

Histological analysis was used to evaluate the pathological changes in the nasal mucosa of rats in each group. The nasal mucosa was arranged neatly and had no abnormality in the control group, while many eosinophilic granulocytes infiltrated the nasal mucosa, and blood stasis was clearly observed in the model group. The pathological changes were significantly alleviated by baicalin (figure 2).

### 3.3. Effects of baicalin on IgE, histamine, IL-1β, IL-4, IL-6 and TNF-α levels in serum

**Figure 3.**
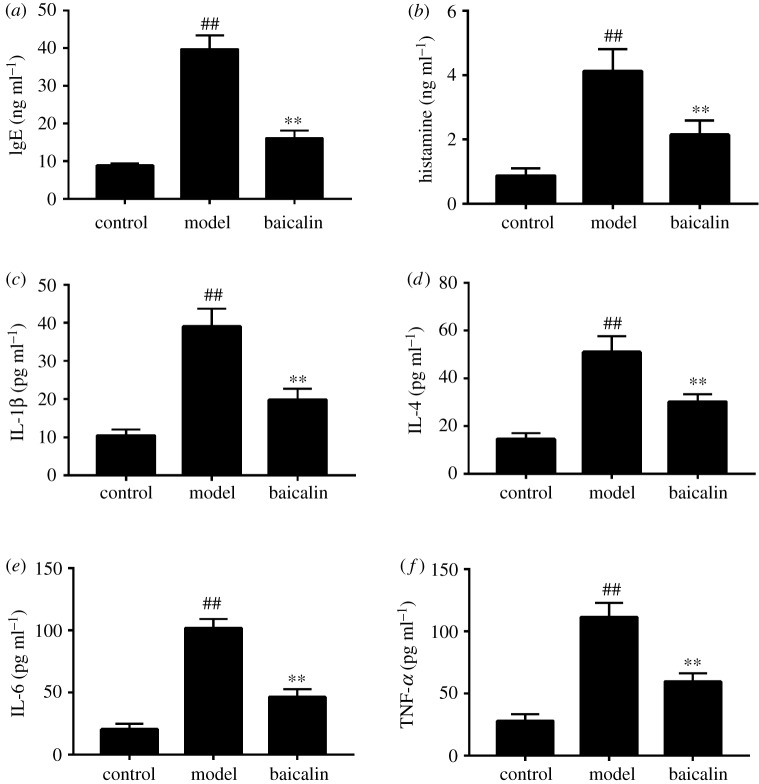
Effects of baicalin on IgE, histamine, IL-1β, IL-4, IL-6 and TNF-α level in serum. (*a*) IgE; (*b*) histamine; (*c*) IL-1β; (*d*) IL-4; (*e*) IL-6; (*f*) TNF-α. The data were presented as mean ± s.d., *n* = 10 per group. Compared to control group, *p < 0.05, **p < 0.01; Compared to model group, #*p* < 0.05, ##*p* < 0.01.

To determine the effect of baicalin on AR models, the levels of IgE, histamine, IL-1β, IL-4, IL-6 and TNF-α were measured by ELISA. The levels of IgE, histamine, IL-1β, IL-4, IL-6 and TNF-α in the model group were markedly increased compared with the control group, and 200 mg kg^−1^ baicalin significantly decreased the levels of IgE, histamine, IL-1β, IL-4, IL-6 and TNF-α in the OVA-sensitized rats (figure 3).

AR is caused by an IgE-mediated inflammatory reaction, including early- and late-phase allergenic mediators [14]. Histamine, as the most iconic indicator of vascular vitality in allergic reactions, is one of the early-phase allergenic mediators; however, histamine as a detection index still has some limitations, mainly reflected in the short half-life [15,16]. Late-phase allergenic mediators, including IL-1β, IL-4, IL-6 and the proinflammatory cytokine TNF-α, can induce IgE synthesis and play an essential role in AR development [17,18].

### 3.4. Metabolomics study

**Figure 4.**
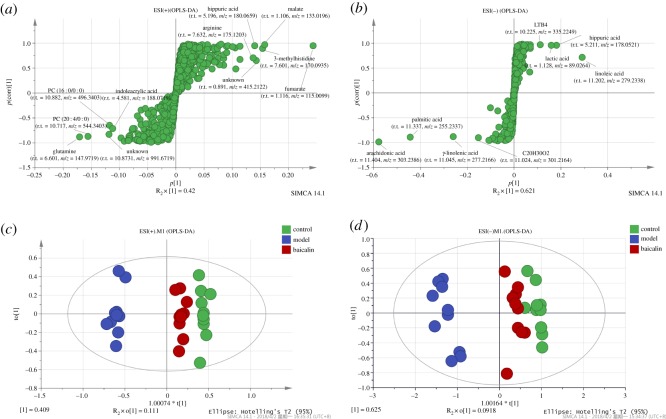
The S-plot analysis and OPLS-DA analysis. (*a*,*b*) The S-plot analysis between control and model groups for the metabolomics data of different ion mode. (*c,d*) The OPLS-DA analysis among control, model and baicalin groups for the metabolomics data of different ion mode.

Reproducibility of the metabolomics approach was determined from six replicated analyses of the same quality control sample (QC) interspersed throughout the analysis. The relative standard deviation (RSD) of the peak area of all metabolites is below 20% (electronic supplemental material, figures S1 and S2), which demonstrates good stability and reproducibility [19]. S-plot analysis was employed by Simca v. 14.1 to determine the specific variation between control and model groups, while variable importance in the projection (VIP) values (VIP greater than 1) of variables were generated and identified using OPLS-DA analysis [20] among control, model and baicalin groups (figure 4). According to the above criteria, 35 metabolites involved in the AR model were selected, and 32 of these were identified based on retention time and accurate elemental compositions with the available databases, such as METLIN (http://metlin.scripps.edu), HMDB (http://www.hmdb.ca) and KEGG (http://genome.jp/kegg) (electronic supplemental material, table S1). The *p*-value was set to 0.05 when determining significantly different metabolites in this study. Next, 30 significantly different metabolites were selected for further study, and 21 metabolites were confirmed with available reference standards by matching their retention time and accurate elemental compositions.

As shown in table 1, the levels of arachidonic acid, palmitic acid, γ-linolenic acid, α-ketoglutaric acid, tetradecanedioic acid, glutamine, PC (20 : 4/0 : 0), indoleacrylic acid, betaine, alanine, serine and PC (16 : 0/0 : 0) were observed to be significantly decreased in the model group (*p* < 0.05). The levels of linoleic acid, hippuric acid, lactic acid, LTB4, citric acid, hydroxyphenyllactic acid, fumarate, malate, 3-methylhistidine, arginine, tyrosine, histamine, N-acetylhistamine, asparagine, valine, LTA4 and creatinine were observed to be markedly increased in the model group (*p* < 0.05). In the baicalin-treated group, the apparent trend of these metabolite levels was the opposite of the model group.

**Table 1.** The information for 30 significantly different metabolites. Compared to control group, *p < 0.05, **p < 0.01; Compared to model group, #*p* < 0.05, ##*p* < 0.01; ‘↑’ means a higher level of metabolites, whereas ‘↓’ represents a lower level of metabolites.

no.	ion mode	r.t. (min)	*m/z*	VIP	elemental composition	metabolites	control group	model group^a^	baicalin group^b^
1	[M − H]^−^	11.404	303.2386	12.75	C_2_0H_32_O_2_	arachidonic acid^c^	747.39 ± 41.24	385.69 ± 21.83**↓	661.68 ± 30.81##↑
2	[M − H]^−^	11.337	255.2337	10.15	C_16_H_32_O_2_	palmitic acid^c^	902.98 ± 17.15	656.12 ± 66.22**↓	842.41 ± 44.67##↑
3	[M − H]^−^	11.202	279.2338	7.39	C_18_H_32_O_2_	linoleic acid	985.79 ± 48.63	1110.77 ± 77.21**↑	1009.47 ± 62.61##↓
4	[M − H]^−^	11.045	277.2166	5.73	C_18_H_30_O_2_	γ-linolenic acid	213.02 ± 29.11	131.22 ± 16.67**↓	193.95 ± 22.25##↑
5	[M − H]^−^	5.211	178.0521	4.03	C_9_H_9_NO_3_	hippuric acid^c^	15.58 ± 2.72	52.38 ± 8.72**↑	25.11 ± 3.01##↓
6	[M − H]^−^	1.128	89.0264	3.48	C_3_H_6_O_3_	lactic acid^c^	3.33 ± 0.23	31.5 ± 6.02**↑	10.13 ± 1.84##↓
7	[M − H]^−^	10.225	335.2249	2.48	C_20_H_32_O_4_	LTB4^c^	2.64 ± 0.3	16.61 ± 2.33**↑	6.03 ± 0.74##↓
8	[M − H]^−^	1.121	145.0139	2.39	C_5_H_6_O_5_	α-ketoglutaric acid^c^	14.95 ± 2.93	1.74 ± 0.31**↓	11.79 ± 2.16##↑
9	[M − H]^−^	1.124	191.0121	1.54	C_6_H_8_O_7_	citric acid^c^	5.24 ± 0.38	10.57 ± 1.59**↑	6.54 ± 0.55##↓
10	[M − H]^−^	4.711	181.0522	1.53	C_9_H_10_O_4_	hydroxyphenyllactic acid	1.77 ± 0.27	7.09 ± 0.89**↑	3.15 ± 0.21##↓
11	[M − H]^−^	9.874	257.1752	1.19	C_14_H_26_O_4_	tetradecanedioic acid	7.58 ± 1.17	4.09 ± 0.65**↓	6.82 ± 0.79##↑
12	[M + H]^+^	1.116	115.0099	9.34	C_4_H_4_O_4_	fumarate^c^	26.15 ± 3.16	68.32 ± 12.58**↑	37.15 ± 3.8##↓
13	[M + H]^+^	6.601	147.9719	6.51	C_4_H_6_NO	glutamine^c^	96.71 ± 8.88	73.69 ± 3.51**↓	91.79 ± 6.43##↑
14	[M + H]^+^	1.106	133.0196	6.03	C_4_H_6_O_5_	malate^c^	9.38 ± 1.7	26.95 ± 3.03**↑	13.95 ± 1.01##↓
15	[M + H]^+^	10.717	544.3403	5.98	C_28_H_50_NO_7_P	PC(20 : 4/0 : 0)	117.21 ± 3.86	97.25 ± 6.23**↓	113.26 ± 3.84##↑
16	[M + H]^+^	7.601	170.0935	5.84	C_7_H_11_N_3_O_2_	3-methylhistidine	39.66 ± 4.71	58.02 ± 4.77**↑	44.71 ± 3.43##↓
17	[M + H]^+^	7.632	175.1203	5.03	C_6_H_14_N_4_O_2_	arginine^c^	115.13 ± 10.11	133.07 ± 10.41**↑	120.81 ± 8.97#↓
18	[M + H]^+^	4.581	188.0719	4.25	C_11_H_9_NO_2_	indoleacrylic acid	82.11 ± 7.27	69.8 ± 5.43**↓	79.77 ± 5.82##↑
19	[M + H]^+^	5.610	182.0825	3.46	C_9_H_11_NO_3_	tyrosine^c^	18.12 ± 1.6	25.16 ± 3.34**↑	20.09 ± 1.34##↓
20	[M + H]^+^	10.590	317.2118	3.07	C_20_H_30_O_3_	LTA4^c^	0.28 ± 0.05	4.77 ± 0.89**↑	1.43 ± 0.21##↓
21	[M + H]^+^	0.912	114.0655	2.58	C_4_H_7_N_3_O	creatinine^c^	11.67 ± 1.21	15.19 ± 0.97**↑	12.68 ± 0.92##↓
22	[M + H]^+^	4.990	132.1023	2.16	C_6_H_13_NO_2_	leucine^c^	5.44 ± 0.96	2.79 ± 0.46**↓	4.81 ± 0.68##↑
23	[M + H]^+^	0.831	118.0859	1.95	C_5_H_11_NO_2_	betaine^c^	24.6 ± 2.98	21.13 ± 2.65**↓	23.95 ± 1.71#↑
24	[M + H]^+^	7.402	112.0888	1.78	C_5_H_9_N_3_	histamine^c^	0.17 ± 0.03	1.7 ± 0.29**↑	0.56 ± 0.08##↓
25	[M + H]^+^	4.202	154.0979	1.61	C_7_H_11_N_3_O	N-acetylhistamine	0.43 ± 0.05	1.71 ± 0.3**↑	0.76 ± 0.08##↓
26	[M + H]^+^	6.102	90.0560	1.58	C_3_H_7_NO_2_	alanine^c^	5.79 ± 0.66	4.27 ± 0.46**↓	5.46 ± 0.51##↑
27	[M + H]^+^	6.851	133.0610	1.58	C_4_H_8_N_2_O_3_	asparagine^c^	13.67 ± 0.88	15.48 ± 1.44**↑	14.26 ± 0.83#↓
28	[M + H]^+^	6.801	106.0521	1.42	C_3_H_7_NO_3_	serine^c^	10.1 ± 0.79	8.65 ± 0.79**↓	9.83 ± 0.62##↑
29	[M + H]^+^	5.411	118.0871	1.01	C_5_H_11_NO_2_	valine^c^	1.36 ± 0.13	1.92 ± 0.26**↑	1.52 ± 0.09##↓
30	[M + H]^+^	10.882	496.3403	3.71	C_24_H_50_NO_7_P	PC (16 : 0/0 : 0)	166 ± 4.3	156.03 ± 6.9**↓	165.07 ± 2.75##↑

^a^Compared to control group, *p < 0.05, **p < 0.01.

^b^Compared to model group, #*p* < 0.05, ##*p* < 0.01.

^c^Confirmed with authentic standards.

**Figure 5.**
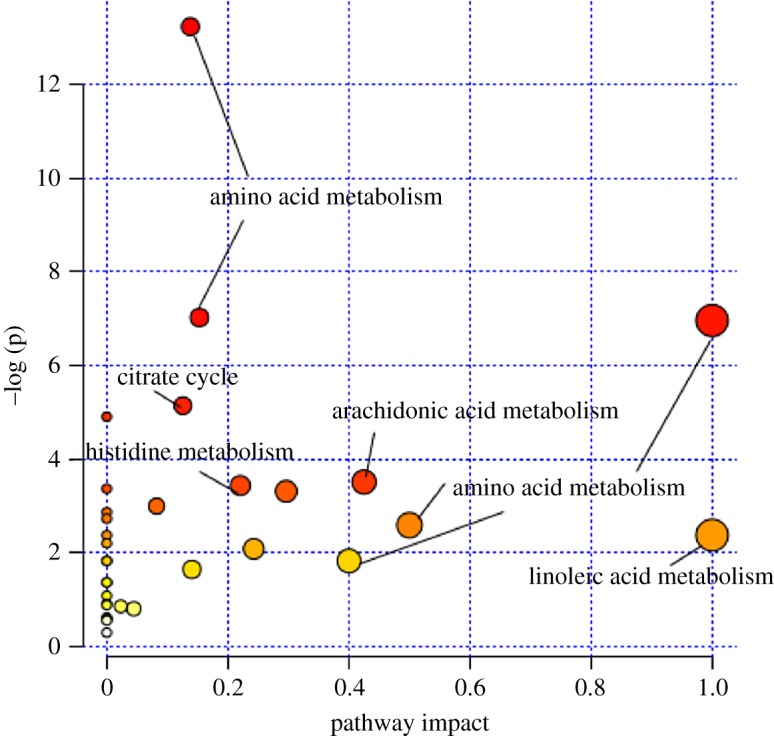
The metabolic pathway of the altered metabolites analysed by MetaboAnalyst 4.0.

MetaboAnalyst 4.0 (http://www.metaboanalyst.ca) was used to analyse metabolic pathways and showed that several pathways, such as arachidonic acid metabolism, citrate cycle, histidine metabolism, linoleic acid metabolism and other amino metabolism, were disordered in the AR model group or in the baicalin group (figure 5).

Several altered metabolites are of special interest because they are directly related to allergic diseases. For instance, linoleic acid (VIP = 7.39), which can induce red blood cells and haemoglobin damage via oxidative mechanisms, is a critical component of polyunsaturated fatty acids [21] and can be converted into γ-linolenic acid (VIP = 5.73) and arachidonic acid (VIP = 12.75). The decreased level of γ-linolenic acid and elevated levels of linoleic acid and arachidonic acid in the model group indicated that linoleic acid metabolism was disordered, and this disorder can be changed by baicalin treatment. Arachidonic acid can generate LTA4 (VIP = 3.07) and LTB4 (VIP = 2.48) via the 5-lipid oxidase pathway. In this study, we observed that LTA4 and LTB4 were increased, followed by arachidonic acid, which is in accordance with this theory [22]. LTB4, synthesized by LTA4 hydrolase from LTA4, is a leukotriene involved in inflammation that plays an important role in the development of AR [23]. These results indicated that arachidonic acid metabolism was also disordered in our study, and with high VIP, arachidonic acid might be used as a specific candidate biomarker for AR. Histamine (VIP = 1.78) was detected by ELISA and was also identified and quantified by UPLC–Q-TOF–MS/MS. Moreover, N-acetylhistamine (VIP = 1.61) and 3-methylhistidine (VIP = 5.84) generated from histamine were also identified and quantified. Interestingly, histamine was described as the ‘gold standard’ for allergic reactions [24], but in this study, the VIP value of histamine was not specific, which may be related to the short half-life of histamine [18]. By contrast, 3-methylhistidine with high VIP may be a new indicator for early-phase allergenic mediators.

Furthermore, a large number of amino acids were significantly altered in AR. Our results suggested that rats sensitized by OVA may consume more energy in order to fight AR when compared to normal rats. Abnormal changes, such as fumarate, malate and α-ketoglutaric acid, belonging to the citric acid cycle, confirm this view. The citric acid cycle, as the linkage among sugar, lipids and amino acid metabolism, is the most important energy source for the body [25]. In addition, other amino acids are substrates for metabolic energy, which could be used for the synthesis of specific proteins, peptides and other nitrogenous compounds [26]. In addition, the changes in PC (20 : 4/0 : 0) and PC (16 : 0/0 : 0) levels, as the precursor of lysoPC, which has been confirmed to be a chemo-attractant for T lymphocytes, showed that the OVA-sensitized rats are in an inflammation state [27].

A similar study of AR treated by baicalin was recently published by another group using guinea pigs. The study focused on IgE, histamine and inflammatory cytokines, and the results suggested that baicalin may be a useful drug in the treatment of AR, which was the same as our results. Additionally, the index IL-8 was replaced with IL-4 in this study, and a randomized double-blind trial with AR showed that IL-4 is important in regulating IgE-mediated allergy [28,29]. IL-4 could also significantly amplify itching symptoms such as histamine [30]; therefore, IL-4 is more closely associated with AR. In addition, the metabolic mechanism of AR and the systematic mechanism of baicalin against OVA-sensitized AR were explored in this study. Other allergy mediators that are specific to AR should be detected and identified, and the different time period mechanisms of baicalin on AR need to be further studied.

## 4. Conclusion

Our study indicated that baicalin has a protective effect on rats with AR by inhibiting the release of inflammatory mediators. Furthermore, metabolomics was applied to systematically study the AR induced by OVA and the mechanism of baicalin on AR. We conclude that baicalin is anti-AR not only because it suppressed the production of inflammatory mediators but also because it regulated arachidonic acid metabolism, the citric acid cycle, histidine metabolism, linoleic acid metabolism and other amino acid metabolism.

## Supplementary Material

Supplemental Table 1

Reviewer comments

## Supplementary Material

Supplemental Figure 1

## Supplementary Material

Supplemental Figure 2

## Supplementary Material

ESI(+)metabolites-all

## Supplementary Material

ESI(-)metabolites-all

## Data accessibility

The datasets supporting this article have been uploaded as part of the electronic supplementary material.

## References

[1] Yamauchi Y et al. 2009 Analysis of the comorbidity of bronchial asthma and allergic rhinitis by questionnaire in 10,009 patients. Allergol. Int. 58, 55–61. (10.2332/allergolint.08-OA-0004) 19050378

[2] Zhou YJ, Wang H, Sui HH, Li L, Zhou CL, Huang JJ 2016 Inhibitory effect of baicalin on allergic response in ovalbumin-induced allergic rhinitis guinea pigs and lipopolysaccharide-stimulated human mast cells. Inflamm. Res. 65, 603–612. (10.1007/s00011-016-0943-0) 27043920

[3] Kakli HA, Riley TD 2016 Allergic rhinitis. Prim. Care 43, 465–475. (10.1016/j.pop.2016.04.009) 27545735

[4] Lee JA, Jang S, Jun JH, Lee MS, Lee E, Kim N, Lee DH 2018 Herbal medicine (Bojungikki-tang) for allergic rhinitis. Medicine (Baltimore), 97, e9551 (10.1097/MD.0000000000009551) 29504976 PMC5779745

[5] Strachan D et al. 1997 Worldwide variations in prevalence of symptoms of allergic rhinoconjunctivitis in children: the International Study of Asthma and Allergies in Childhood (ISAAC). Pediatr. Allergy Immunol. 8, 161–168. (10.1111/j.1399-3038.1997.tb00156.x) 9553981

[6] Settipane RA 2001 Demographics and epidemiology of allergic and nonallergic rhinitis. In Allergy and asthma proceedings, vol. 22, pp. 185–189. Providence, RI: OceanSide Publications. 11552666

[7] Licari A, Ciprandi G, Marseglia A, Castagnoli R, Barberi S, Caimmi S, Marseglia GL 2014 Current recommendations and emerging options for the treatment of allergic rhinitis. Expert Rev. Clin. Immunol. 10, 1337–1347. (10.1586/1744666X.2014.955476) 25225773

[8] Marple BF 2010 Allergic rhinitis and inflammatory airway disease: interactions within the unified airspace. Am. J. Rhinol. Allergy 24, 249–254. (10.2500/ajra.2010.24.3499) 20819460

[9] Eifan AO, Durham SR 2016 Pathogenesis of rhinitis. Clin. Exp. Allergy 46, 1139–1151. (10.1111/cea.12780) 27434218

[10] Xue CC, Thien FC, Zhang JJ, Da Costa C, Li CG 2003 Treatment for seasonal allergic rhinitis by Chinese herbal medicine: a randomized placebo controlled trial. Altern. Ther. Health Med. 9, 80–87. 14526714

[11] Xu L, Li J, Zhang Y, Zhao P, Zhang X 2017 Regulatory effect of baicalin on the imbalance of Th17/Treg responses in mice with allergic asthma. J. Ethnopharmacol. 208, 199–206. (10.1016/j.jep.2017.07.013) 28709893

[12] Li C et al. 2017 Mesenchymal stromal cells ameliorate acute allergic rhinitis in rats. Cell Biochem. Funct. 35, 420–425. (10.1002/cbf.3291) 28940415 PMC5698748

[13] Xu Y, Dou D, Ran X, Liu C, Chen J 2015 Integrative analysis of proteomics and metabolomics of anaphylactoid reaction induced by Xuesaitong injection. J. Chromatogr. A 1416, 103–111. (10.1016/j.chroma.2015.09.019) 26372445

[14] Lv C, Zhang Y, Shen L 2018 Preliminary clinical effect evaluation of resveratrol in adults with allergic rhinitis. Int. Arch. Allergy Immunol. 175, 231–236. (10.1159/000486959) 29539616

[15] Hashimoto T, Ohata H, Honda K 2006 Lysophosphatidic acid (LPA) induces plasma exudation and histamine release in mice via LPA receptors. J. Pharmacol. Sci. 100, 82–87. (10.1254/jphs.FPJ05030X) 16404130

[16] Kun T, Jakubowski L 2012 Influence of MRI contrast media on histamine release from mast cells. Pol. J. Radiol. 77, 19–24. (10.12659/PJR.883370) PMC3447429 23049577

[17] Shen Y, Ke X, Yun L, Hu GH, Kang HY, Hong SL 2018 Decreased expression of interleukin-37 and its anti-inflammatory effect in allergic rhinitis. Mol. Med. Rep. 17, 1333–1339. (10.3892/mmr.2017.7988) 29115624

[18] Youssef LA, Schuyler M, Wilson BS, Oliver JM 2010 Roles for the high affinity IgE receptor, FcεRI, of human basophils in the pathogenesis and therapy of allergic asthma: disease promotion, protection or both?. Open Allergy J. 3, 91–101. (10.2174/1874838401003010091) 25018787 PMC4090948

[19] Cui L, Lu H, Lee YH 2018 Challenges and emergent solutions for LC-MS/MS based untargeted metabolomics in diseases. Mass Spectrom. Rev. 37, 772–792. (10.1002/mas.21562) 29486047

[20] Yang QJ et al. 2018 Serum and urine metabolomics study reveals a distinct diagnostic model for cancer cachexia. J Cachexia Sarcopenia Muscle 9, 71–85. (10.1002/jcsm.12246) 29152916 PMC5803608

[21] Yuan T, Fan WB, Cong Y, Xu HD, Li CJ, Meng J, Bao NR, Zhao JN 2015 Linoleic acid induces red blood cells and hemoglobin damage via oxidative mechanism. Int. J. Clin. Exp. Pathol. 8, 5044–5052. 26191198 PMC4503070

[22] Hsu CH, Hu CM, Lu KH, Yang SF, Tsai CH, Ko CL, Sun HL, Lue KH 2012 Effect of selective cysteinyl leukotriene receptor antagonists on airway inflammation and matrix metalloproteinase expression in a mouse asthma model. Pediatr. Neonatol. 53, 235–244. (10.1016/j.pedneo.2012.06.004) 22964281

[23] Qiu Q, Xu M, Lu C, Chen J, Chen S, Kong W, Han H 2016 Safety and efficacy of rush allergen-specific immunotherapy in Chinese allergic rhinitis patients. Int. J. Immunopathol. Pharmacol. 29, 720–725. (10.1177/0394632016659301) 27510816 PMC5806822

[24] Xu Y, Kang T, Dou D, Kuang H 2015 The evaluation and optimization of animal model for anaphylactoid reaction induced by injections. Asian Pac. J. Allergy Immunol. 33, 330–338. (10.12932/AP0619.33.4.2015) 26708398

[25] Song H, Li WL, Liu BM, Sun XM, Ding JX, Chen N 2017 Study of the estrogenic-like mechanism of glycosides of cistanche using metabolomics. RSC Adv 7, 39 403–39 410. (10.1039/C7RA06930H)

[26] Grillo MA, Lanza A, Colombatto S 2008 Transport of amino acids through the placenta and their role. Amino Acids 34, 517–523. (10.1007/s00726-007-0006-5) 18172742

[27] Lu X, Lian X, Zheng J, Ai N, Ji C, Hao C 2016 LC-ESI-TOF-MS-based metabolomic analysis of ginsenoside Rd-induced anaphylactoid reaction in mice. RSC Adv 6, 19 545–19 554. (10.1039/C5RA24301G)

[28] Appel K, Munoz E, Navarrete C, Cruz-Teno C, Biller A, Thiemann E 2018 Immunomodulatory and inhibitory effect of Immulina^®^, and Immunloges^®^ in the Ig-E mediated activation of RBL-2H3 cells. A new role in allergic inflammatory responses. Plants (Basel) 7, 13 (10.3390/plants7010013) PMC5874602 29495393

[29] Mao TK, Van de Water J, Gershwin ME 2005 Effects of a *Spirulina*-based dietary supplement on cytokine production from allergic rhinitis patients. J. Med. Food 8, 27–30. (10.1089/jmf.2005.8.27) 15857205

[30] Oetjen LK et al. 2017 Sensory neurons co-opt classical immune signaling pathways to mediate chronic itch. Cell 171, 217–228.e13. (10.1016/j.cell.2017.08.006) 28890086 PMC5658016

