# Supplementary material for: Metabolomics analysis of baicalin on ovalbumin-sensitized allergic rhinitis rats
Source: R Soc Open Sci. 2019 Feb 20;6(2):181081. doi: 10.1098/rsos.181081 (PMC6408364; doi:10.1098/rsos.181081)
Supplement: Supplemental Table 1 [file rsos181081supp1.docx]

**Supplemental Table1. The metabolites altered according to the S-plot analysis and VIP value**

| No. | Ion mode | r.t. (min) | m/z | VIP | Elemental composition | Metabolites |
| --- | --- | --- | --- | --- | --- | --- |
| 1 | [M-H]^-^ | 11.404 | 303.2386 | 12.75 | C_20_H_32_O_2_ | arachidonic acid |
| 2 | [M-H]^-^ | 11.337 | 255.2337 | 10.15 | C_16_H_32_O_2_ | Palmitic acid |
| 3 | [M-H]^-^ | 11.202 | 279.2338 | 7.39 | C_18_H_32_O_2_ | Linoleic acid |
| 4 | [M-H]^-^ | 11.045 | 277.2166 | 5.73 | C_18_H_30_O_2_ | γ-Linolenic acid |
| 5 | [M-H]^-^ | 5.211 | 178.0521 | 4.03 | C_9_H_9_NO_3_ | hippuric acid |
| 6 | [M-H]^-^ | 1.128 | 89.0264 | 3.48 | C_3_H_6_O_3_ | lactic acid |
| 7 | [M-H]^-^ | 10.225 | 335.2249 | 2.48 | C_20_H_32_O_4_ | LTB4 |
| 8 | [M-H]^-^ | 1.121 | 145.0139 | 2.39 | C_5_H_6_O_5_ | α- ketoglutaric acid |
| 9 | [M-H]^-^ | 1.124 | 191.0121 | 1.54 | C_6_H_8_O_7_ | citric acid |
| 10 | [M-H]^-^ | 4.711 | 181.0522 | 1.53 | C_9_H_10_O_4_ | Hydroxyphenyllactic acid |
| 11 | [M-H]^-^ | 9.874 | 257.1752 | 1.19 | C_14_H_26_O_4_ | Tetradecanedioic acid |
| 12 | [M-H]^-^ | 11.024 | 301.2164 | 3.37 | C_20_H_30_O_2_ | unknown |
| 13 | [M+H]^+^ | 1.116 | 115.0099 | 9.34 | C_4_H_4_O_4_ | Fumarate |
| 14 | [M+H]^+^ | 6.601 | 147.9719 | 6.51 | C_4_H_6_NO | glutamine |
| 15 | [M+H]^+^ | 1.106 | 133.0196 | 6.03 | C_4_H_6_O_5_ | Malate |
| 16 | [M+H]^+^ | 10.717 | 544.3403 | 5.98 | C_28_H_50_NO_7_P | PC(20:4/0:0) |
| 17 | [M+H]^+^ | 7.601 | 170.0935 | 5.84 | C_7_H_11_N_3_O_2_ | 3-Methylhistidine |
| 18 | [M+H]^+^ | 7.632 | 175.1203 | 5.03 | C_6_H_14_N_4_O_2_ | arginine |
| 19 | [M+H]^+^ | 4.581 | 188.0719 | 4.25 | C_11_H_9_NO_2_ | Indoleacrylic acid |
| 20 | [M+H]^+^ | 5.610 | 182.0825 | 3.46 | C_9_H_11_NO_3_ | tyrosine |
| 21 | [M+H]^+^ | 10.590 | 317.2118 | 3.07 | C_20_H_30_O_3_ | LTA4 |
| 22 | [M+H]^+^ | 0.912 | 114.0655 | 2.58 | C_4_H_7_N_3_O | creatinine |
| 23 | [M+H]^+^ | 4.990 | 132.1023 | 2.16 | C_6_H_13_NO_2_ | leucine |
| 24 | [M+H]^+^ | 0.831 | 118.0859 | 1.95 | C_5_H_11_NO_2_ | betaine |
| 25 | [M+H]^+^ | 7.402 | 112.0888 | 1.78 | C_5_H_9_N_3_ | histamine |
| 26 | [M+H]^+^ | 4.578 | 205.0976 | 1.67 | C_11_H_12_N_2_O_2_ | tryptophan |
| 27 | [M+H]^+^ | 4.202 | 154.0979 | 1.61 | C_7_H_11_N_3_O | N-Acetylhistamine |
| 28 | [M+H]^+^ | 6.102 | 90.0560 | 1.58 | C_3_H_7_NO_2_ | alanine |
| 29 | [M+H]^+^ | 6.851 | 133.0610 | 1.58 | C_4_H_8_N_2_O_3_ | asparagine |
| 30 | [M+H]^+^ | 4.986 | 132.1053 | 1.44 | C_6_H_13_NO_2_ | isoleucine |
| 31 | [M+H]^+^ | 6.801 | 106.0521 | 1.42 | C_3_H_7_NO_3_ | serine |
| 32 | [M+H]^+^ | 5.411 | 118.0871 | 1.01 | C_5_H_11_NO_2_ | valine |
| 33 | [M+H]^+^ | 10.882 | 496.3403 | 3.71 | C_24_H_50_NO_7_P | PC(16:0/0:0) |
| 34 | [M+H]^+^ | 10.873 | 991.6719 | 4.55 | - | unknown |
| 35 | [M+H]^+^ | 0.891 | 415.2122 | 5.31 | - | unknown |
