# Supplementary figures and images for: Metabolomics analysis of baicalin on ovalbumin-sensitized allergic rhinitis rats
Source: R Soc Open Sci. 2019 Feb 20;6(2):181081. doi: 10.1098/rsos.181081 (PMC6408364; doi:10.1098/rsos.181081)

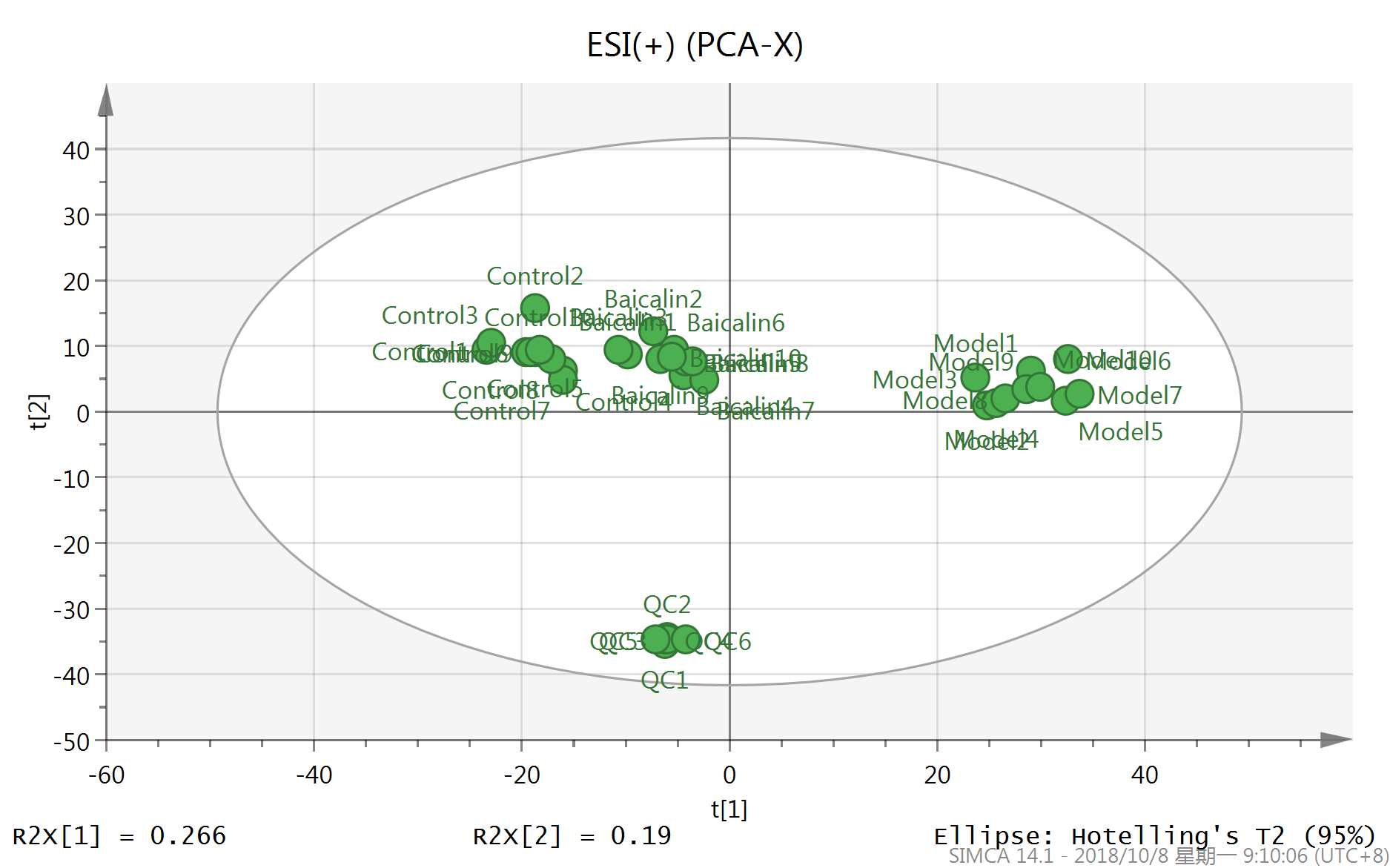

Supplement: Supplemental Figure 1 [file rsos181081supp2.tif]

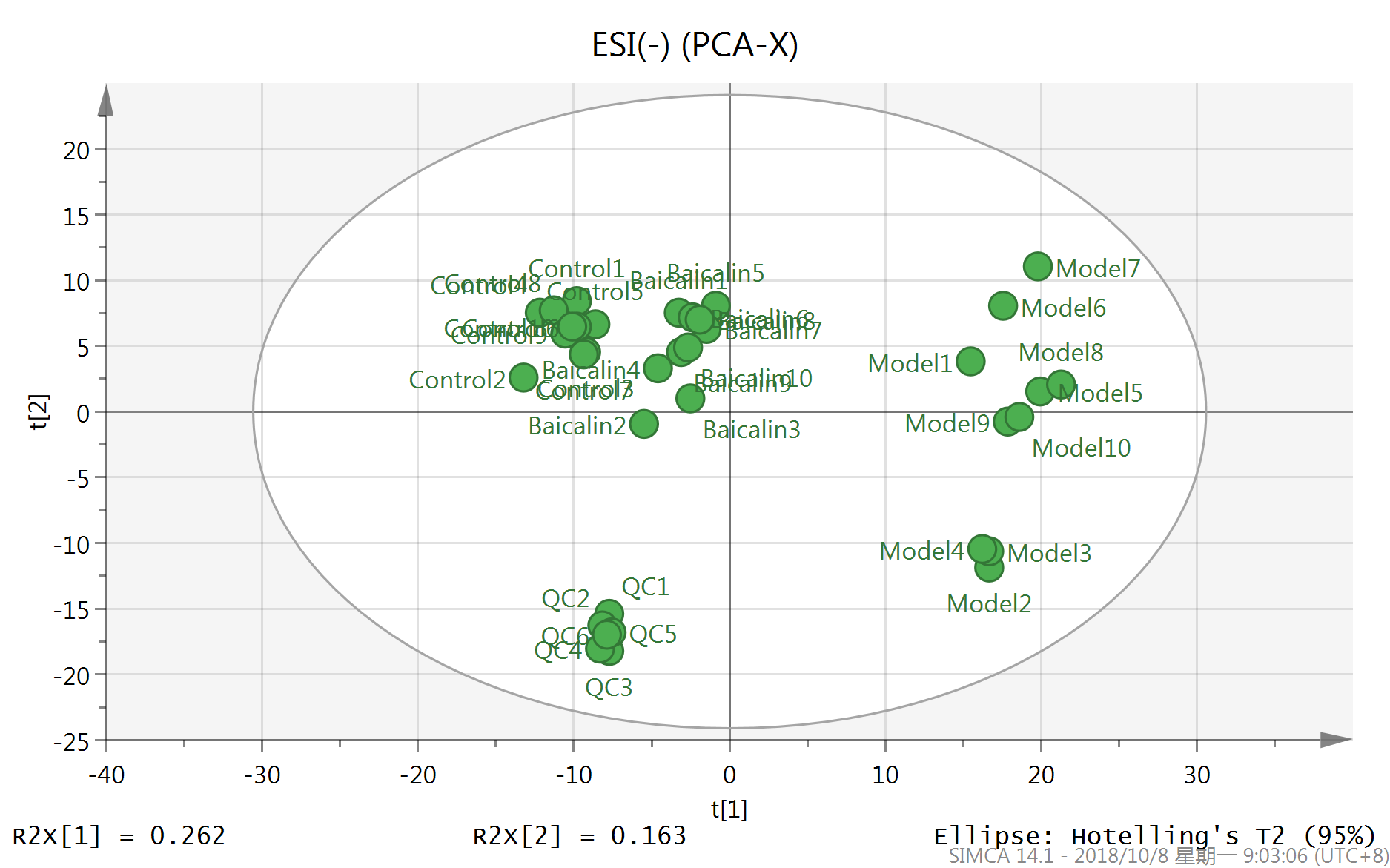

Supplement: Supplemental Figure 2 [file rsos181081supp3.tif]
